# Clearing asymptomatic parasitaemia increases the specificity of the definition of mild febrile malaria

**DOI:** 10.1016/j.vaccine.2007.07.057

**Published:** 2007-11-23

**Authors:** Philip Bejon, Tabitha Mwangi, Brett Lowe, Norbert Peshu, Adrian V.S. Hill, Kevin Marsh

**Affiliations:** a Kenya Medical Research Institute, Centre for Geographical Medicine Research (Coast), Kenya; b Centre for Clinical Vaccinology and Tropical Medicine, University of Oxford, Oxford OX3 7LJ, United Kingdom; c Wellcome Trust Centre for Human Genetics, University of Oxford, Roosevelt Drive, Oxford OX3 7BN, United Kingdom; d Nuffield Department of Clinical Medicine, Oxford University, John Radcliffe Hospital, Oxford, United Kingdom

**Keywords:** *Plasmodium falciparum*, Malaria attributable fraction, Specificity, Febrile malaria, Definition

## Abstract

In clinical trials, the specificity of the disease endpoint is critical to an accurate estimate of vaccine efficacy. We used a logistic regression model to analyse parasite densities among children before and after treatment with antimalarials, in order to estimate the impact that clearing asymptomatic parasitaemia had on the specificity of the endpoint of febrile malaria. The malaria attributable fever fraction was higher after antimalarial treatment (i.e. fever and parasitaemia were more likely to be causally related), implying that drug treatment prior to monitoring decreased the misclassification of febrile malaria. In intervention studies with febrile malaria as an endpoint, clearing asymptomatic parasitaemia increases the study's power more effectively than raising the threshold parasitaemia.

## 1 Introduction

Field trials of candidate malaria vaccines often use febrile malaria as an endpoint [1–4]. The case definition of febrile malaria usually requires a measured temperature and parasitaemia. In endemic areas non-malarial fever is common in young children, and by chance frequently coincides with asymptomatic parasitaemia [5]. Including these cases in the endpoint thus reducing the specificity of the case definition for febrile malaria. It is possible to increase the specificity by adopting a threshold parasitaemia, on the basis that asymptomatic parasitaemia tends to be at a lower density than febrile malaria. However, since the distributions of asymptomatic parasitaemia density and febrile malaria density overlap, this threshold is necessarily a compromise between sensitivity and specificity. In epidemiological studies and clinical trials both sensitivity and specificity are desirable to maximise the power of the study [6–8].

### 1.1 Clearing asymptomatic parasitaemia

Treatment of asymptomatic parasitaemia is done for various reasons. It is an integral part of the strategy of intermittent preventative treatment [9]. In vaccine trials with the primary endpoint of time to infection, pre-surveillance clearance of asymptomatic infection is essential [10,11]. Where the primary endpoint is febrile or severe disease this would not be necessary, and some data suggest that efficacy might be better measured without pre-treatment [4]. Since asymptomatic parasitaemia complicates the definition of febrile malaria, it is possible that clearing asymptomatic parasitaemia might alter the case definition of febrile malaria, and may result in a more specific definition without any loss in sensitivity.

### 1.2 Malaria attributable fever fractions

The malaria attributable fever fraction (MAFF) is defined as the fraction of fevers that can be attributed to malaria infection (i.e. the remainder of fevers outside this fraction are the result of coincident non-malarial fever and asymptomatic parasitaemia). While it may not be possible to determine which individuals are “true” cases of febrile malaria, and so calculate this fraction by an arithmetically calculated numerator and denominator, using a logistic regression model allows the MAFF in a population to be estimated [12]. This method fits parasite density to the probability of febrile malaria in a logistic regression model. Once MAFFs have been calculated at different ranges of parasitaemia, it is then possible to calculate the sensitivity and specificity of different parasite thresholds. This has been used to generate case definitions in different age groups and malaria transmission intensities [13–15]. Here, we used this approach to examine the hypothesis that clearing asymptomatic parasitaemia would increase the specificity of the case definition of clinical malaria in a cohort of children under longitudinal surveillance.

### 1.3 The impact of specificity of the endpoint

The specificity of the endpoint is critical to a trial's ability to correctly estimate vaccine efficacy. If a vaccine is efficacious against febrile malaria, but not against asymptomatic parasitaemia, then including cases of non-malarial fever that coincide with asymptomatic parasitaemia in the endpoint will reduce the apparent efficacy of the vaccine. Our objective in this analysis was to compare infections before and after parasite clearance was given, and so calculate the MAFF, sensitivity, and specificity at various parasite densities. This then allows the relative power of studies with and without drug clearance to be estimated.

## 2 Methods

### 2.1 Study participants

Four hundred and five children, aged 1–6-years-old (inclusive), were randomised for either an experimental prime boost malaria vaccine or control vaccination (rabies). The children were healthy, and resident in Junju, within Kilifi District, a rural area of coastal Kenya. The process of randomization and vaccination is detailed elsewhere [3]. Since the vaccination was not efficacious, no adjustment has been made for vaccination group.

### 2.2 Blood samples and clearance of parasitaemia

Cross-sectional surveys to examine peripheral blood parasitaemia were conducted at recruitment (January 2005) before any antimalarial treatment was given. After three vaccinations and antimalarial treatment, cross-sectional surveys were conducted 2 weeks after antimalarials were given (May 2005), then after 3 months in September 2005 (end of the rainy season) and after 8 months in January 2006 (during the dry season). Antimalarial treatment to clear asymptomatic parasitaemia was given 1 week after the final vaccination, using 7 days of directly observed dihydroartemisinin monotherapy (2 mg/kg on the first day, followed by 1 mg/kg for 6 days). Four hundred and five children received at least one vaccination, and of these 387 finished the vaccination course and completed at least one further monitoring visit for malaria episodes. Three hundred and sixty children complied with the 7 days observed artesunate and were parasite negative 2 weeks later. Analysis here is restricted to these 360 children. Ninety-four fevers were assessed in the 3 months before clearance of asymptomatic parasitaemias treatment, and 353 in the 9 months after clearance. Further cross-sectional surveys were taken 3 months post-drug treatment (early August 2005) and 9 months post-drug treatment (January 2006).

### 2.3 Monitoring for malaria episodes

Children were seen weekly by field workers, and blood films made when the temperature was >37.5°. Field workers lived in the study area, and parents brought their children for assessment between regular weekly visits if the child developed fever. Treatment for episodes of malaria was with the Government of Kenya recommended first line treatment, artemether–lumefantrine. When the mother reported the child was hot, but an objectively elevated temperature was not identified, blood films and rapid testing was not performed, but the field worker returned to the child a further three times in the next 24 h. Rapid testing and blood films were performed if the temperature was elevated on any of these visits. Parents brought their children for assessment in between regular weekly visits if they thought the child had developed fever, and the child was assessed as above. Field workers were recruited from the villages in which the study was conducted, and so were readily accessible to the parents.

### 2.4 Laboratory procedures

Blood films were examined in duplicate by two microscopists, and examined a third time if there was a discrepancy. The results of blood films at cross-sectional bleeds were not immediately available, and asymptomatic parasitaemic children were not treated unless they developed fever.

### 2.5 Analysis

The parasite densities from the cross-sectional survey and episodes of febrile malaria before drug treatment were used in a logistic model to relate parasite density to the likelihood of fever. The data are analysed by three time periods. The pre-treatment period included 6–7 weeks of dry weather and 6–7 weeks of rains. The post-treatment period was analysed separately for the first 12 weeks of rains, and for the subsequent 24 weeks of dry weather. The pre-treatment analysis used malaria episodes between January 2005 and May 2005, and the cross-sectional bleed from January 2005. Vaccinations were given between January and May 2005, and antimalarial drug treatment given in May 2005. The post-treatment rainy season analysis used malaria episodes between May 2005 and September 2005, and the cross-sectional bleed from September 2005. The post-treatment dry season analysis used malaria episodes between September 2005 and January 2006, and the cross-sectional bleed from January 2006.

The method for deriving malaria attributable fever fractions (MAFFs) has been described previously [5]. Briefly, the logistic regression model is used (logit(*p*) = *a* + *bx*^*τ*^, where *p* is the probability of fever, *x* the density of parasitaemia and *τ* is the power function of the parasite density which maximises the likelihood estimation for the different time points). The coefficient *b* can then be used to estimate the risk of febrile malaria in individual children, and the average risk of malaria among a group of children with fever gives the fraction of those children whose fever was a direct result of malaria (i.e. the malaria attributable fever fraction, MAFF). This calculation can be repeated after selecting children above a threshold parasitaemia, and used to calculate the sensitivity and specificity of threshold values for parasite density.

Separate analyses were conducted by vaccination status. MAFFs were not different by vaccination group, and vaccination had no effect on frequency of malaria episodes or parasitaemia [3]. The combined analysis is presented here.

## 3 Results

In the pre-treatment cross-sectional survey (January 2005), 71% of children were parasitaemic. 2 weeks after drug treatment 2% were parasitaemic, but 3 months (September 2005, post-treatment rainy season) and 9 months later (January 2006, post-treatment dry season), 27% and 33% of children were parasitaemic, respectively.

### 3.1 Parasitaemias during febrile episodes varied before and after drug clearance

**Fig. 1:**
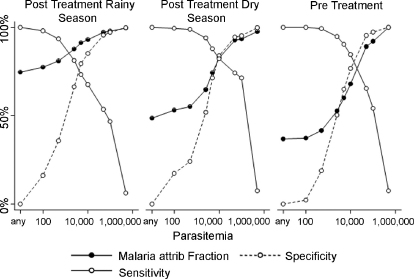
The sensitivity, specificity and malaria attributable fever fraction curves for varying parasitaemia thresholds are shown for subjects before parasite clearance, and for rainy and dry season follow up after parasite clearance. There is little difference positive predictive values of positive films are consistently higher after treatment.

Before drug clearance of asymptomatic parasitaemia, 47 of 121 (38%) of children with fever had no parasitaemia, compared with 75 of 182 (41%) during the rainy season post-drug clearance (Fig. 1), and 70 of 124 (56%) in the next 24 weeks of the dry season post-treatment.

### 3.2 Specificity, sensitivity and malaria attributable fractions

A logistic model was fitted to the data as described previously [5]. The sensitivity, specificity and malaria attributable fever fractions (MAFFs) given by using different parasitaemia thresholds are shown in Fig. 1. Although sensitivity and specificity curves generated by the model were similar, the MAFFs calculated among all children with positive blood films were greater after curative treatment, particularly at lower parasitaemia thresholds.

Pre-treatment, there were 74 febrile parasitaemic children over 12 weeks of monitoring. An MAFF of 37% (95% CI 26–48%) among all parasitaemic children suggests that there were only 27 true episodes of malaria among these children (i.e. 2.3 week^−1^).

Malaria transmission was highest during the post-treatment rainy season. There were 107 febrile parasitaemic children during 12 weeks, and since the MAFF (for all children with parasitaemia) was 74% (95% CI 63–83%) this suggests 80 true episodes of malaria (i.e. 6.7 week^−1^).

During the 24-week post-treatment dry season, there were 49 febrile parasitaemic children, and an MAFF (for all children with parasitaemia) of 51% (95% CI 37–63%) suggests that 24 had true episodes of malaria (i.e. 1 week^−1^).

Thus, the MAFF for all parasitaemia children during the post-treatment dry season, 51% (95% CI 37–63%), was intermediate between the MAFF during the pre-treatment period, at 37% (95% CI 26–48%), and the MAFF seen during the post-treatment rainy season, at 74% (95% CI 63–83%).

Using a threshold of 2500 paras/μl, the MAFFs were 52% (CI 35–69%), 87% (CI 73–87%) and 65% (CI 50–80%) pre-treatment, post-treatment rainy season and post-treatment dry season, respectively.

## 4 Discussion

A phase 2b randomized controlled trial to measure the efficacy of a candidate malaria vaccine was conducted. In common with many phase 2b trials in children, the primary endpoint was episodes of febrile malaria. The specificity of the endpoint is critical to the accuracy of estimates of efficacy in clinical trials. Curative drug treatment before monitoring was used to clear asymptomatic parasitaemia before vaccination was complete. It is unclear whether this is necessary in vaccine studies, and there are recommendations to not do so in studies of blood stage vaccines [16]. In this analysis we have examined the impact this had on case definitions.

### 4.1 Malaria attributable fever fractions varied following treatment

The logistic regression model (Fig. 1) suggested that before drug clearance the malaria attributable fever fractions (MAFFs) were low. A threshold of 50,000 paras/μl would be required for a case definition with a 90% MAFF pre-treatment. For a threshold of 2500 paras/μl, the MAFF was only 52%. Post-treatment, during the rainy season, the MAFFs increased, to 75% for all parasitaemic children, and 87% above a threshold of 2500 paras/μl. However, there was more intense malaria transmission during this time period (6.7 cases/week versus 2.3 cases/week pre-treatment). It is difficult to judge how much of the increase in the case definition is due to rising malaria transmission, and how much due to the treatment of asymptomatic parasitaemia. However, malaria transmission was very low during the post-treatment dry season, at 1 case/week. This was associated with a fall in MAFFs, but not to the pre-treatment levels. Although the difference between post-treatment rainy and dry season shows that transmission intensity influences the MAFF, transmission was higher during pre-treatment than it was for post-treatment dry season (2.3 cases/week versus 1 case/week). Despite a much higher transmission intensity pre-treatment, the post-treatment dry season MAFFs were higher. Thus, both transmission intensity and the frequency of asymptomatic parasitaemia influenced the MAFFs.

The sensitivities and specificity plots against parasitaemia were similar before and after parasite clearance (Fig. 1), even though the MAFF among children with positive blood films varied considerably. Sensitivity and specificity are properties intrinsic to the performance of the test, but the MAFF depends on sensitivity, specificity and the pre-test probability. The pre-test probability that parasitaemia indicated febrile malaria, and not asymptomatic parasitaemia, had been altered by drug clearance. The higher MAFF increases the accuracy and power of the study after clearance.

### 4.2 Predicted impact on trial results

A standard power calculation suggests that if vaccine efficacy was 50%, a trial with 90% power would require 85 subjects in each arm, given the rates of malaria seen here. How can one account for the situation where the MAFF is only 50%? If one assumes that the vaccine prevents fever causally related to parasitaemia, but not non-malaria fever that coincides by chance with asymptomatic parasitaemia, then the apparent vaccine efficacy would be 25%, and the required sample size would be 265 subjects in each arm.

One could introduce a parasitaemia threshold to the case definition, in order to exclude cases of asymptomatic parasitaemia. However, this inevitably excludes some genuine cases of febrile malaria with low parasitaemia, and so the chosen threshold is a compromise between sensitivity and specificity. This then has the effect of increasing the sample size.

In our data, a case definition of any parasitaemia occurred at an incidence of 0.55 per subject, but the MAFF was 36% (i.e. fever was causally related to parasitaemia in 36% of the cases so defined). This would have required 512 subjects per arm to detect 50% protection with 90% power. At 50,000 paras/μl 485 subjects per arm would be required (because of increased specificity). However, higher parasitaemia thresholds do not result in further reductions in sample size, because of the competing effect of lowered sensitivity. At 100,000 paras/μl, 578 subjects per arm are required.

However, after drug clearance, the MAFF rose to 0.64, without any loss in sensitivity. This would require 158 subjects per arm. Optimal power after drug clearance would have been seen using a threshold of only 100 paras/μl (143 subjects per arm) and the sample size required would rise to 217 per arm at 5000 paras/μl.

Models have been used to demonstrate the theoretical underestimates and overestimates of blood stage vaccine efficacy against febrile malaria [14]. Our data suggest that more accurate estimates of efficacy in the field might be obtained following clearance of asymptomatic parasitaemia. In one study of a blood stage vaccine, greater efficacy was observed in the cohort of children without clearance of asymptomatic parasitaemia [4], but this was primarily a reduction in parasite density rather than numbers of febrile episodes. However, intermittent presumptive treatment has been extensively studied [9], and using antimalarials prior to monitoring may reduce subsequent rates of febrile malaria. This impact must be balanced against the possibility of reduced specificity leading to a biased, lower estimate of vaccine efficacy.

In recent studies of an efficacious vaccine [1,17] there was no obvious difference in the point estimates of efficacy using varying parasitaemia thresholds. However, the vaccine had similar efficacy against asymptomatic parasitaemia and febrile malaria. Therefore, a child with non-malarial fever who had received the vaccine would be less likely to have asymptomatic parasitaemia giving rise to a spurious diagnosis of malaria. This explains the consistent measures of vaccine efficacy seen at varying parasite densities, since the vaccinated children had lower rates of “true” febrile malaria and lower rates of “false positive” febrile malaria. In certain scenarios this effect might lead to an overestimate of vaccine efficacy, for instance if a vaccine was efficacious during a rainy season, and monitoring of efficacy then continued during a following dry season. The majority of cases identified during the low transmission of the dry season might actually be coincident non-malarial fever and chronic asymptomatic parasitaemia acquired during the preceding rainy season. If the prevalence of asymptomatic parasitaemia was lower at the start of the dry season among vaccinated children (because of protection against asymptomatic parasitaemia during the rainy season), then the apparent efficacy during ongoing monitoring would be overestimated. It would be predicted that MAFFs would demonstrate this effect, and it is therefore particularly important that MAFFs are calculated separately for intervention groups, and by different time periods if waning efficacy is a possibility.
